# Supplementary material for: Investigating the influence of physiologically relevant hydrostatic pressure on CHO cell batch culture
Source: Sci Rep. 2021 Jan 8;11:162. doi: 10.1038/s41598-020-80576-8 (PMC7794228; doi:10.1038/s41598-020-80576-8)
Supplement: Supplementary file 1 — Supplementary Information 1. [file 41598_2020_80576_MOESM1_ESM.zip › S1/IgG/Feb14 0 30 60 90/30mmHg Jan20 2.pdf]

Data File C:\Users\P...emStation\1\Data\TK_CHO_IgG 2019-02-14 13-34-34\005-4-ML 30 Jan20.D
Sample Name: ML 30 Jan20

===

Acq. Operator : SYSTEM Seq. Line : 5
Sample Operator : SYSTEM
Acq. Instrument : HPLC-0039 Location : 4
Injection Date : 2/14/2019 1:53:00 PM Inj : 1
Inj Volume : 100.000 µl
Sequence File : C:\Users\Public\Documents\ChemStation\1\Data\TK_CHO_IgG 2019-02-14 13-34-34\TK_CHO_IgG.S
Method : C:\Users\Public\Documents\ChemStation\1\Data\TK_CHO_IgG 2019-02-14 13-34-34\CHO_IgG_LC.M (Sequence Method)
Last changed : 2/8/2019 3:17:23 PM by SYSTEM

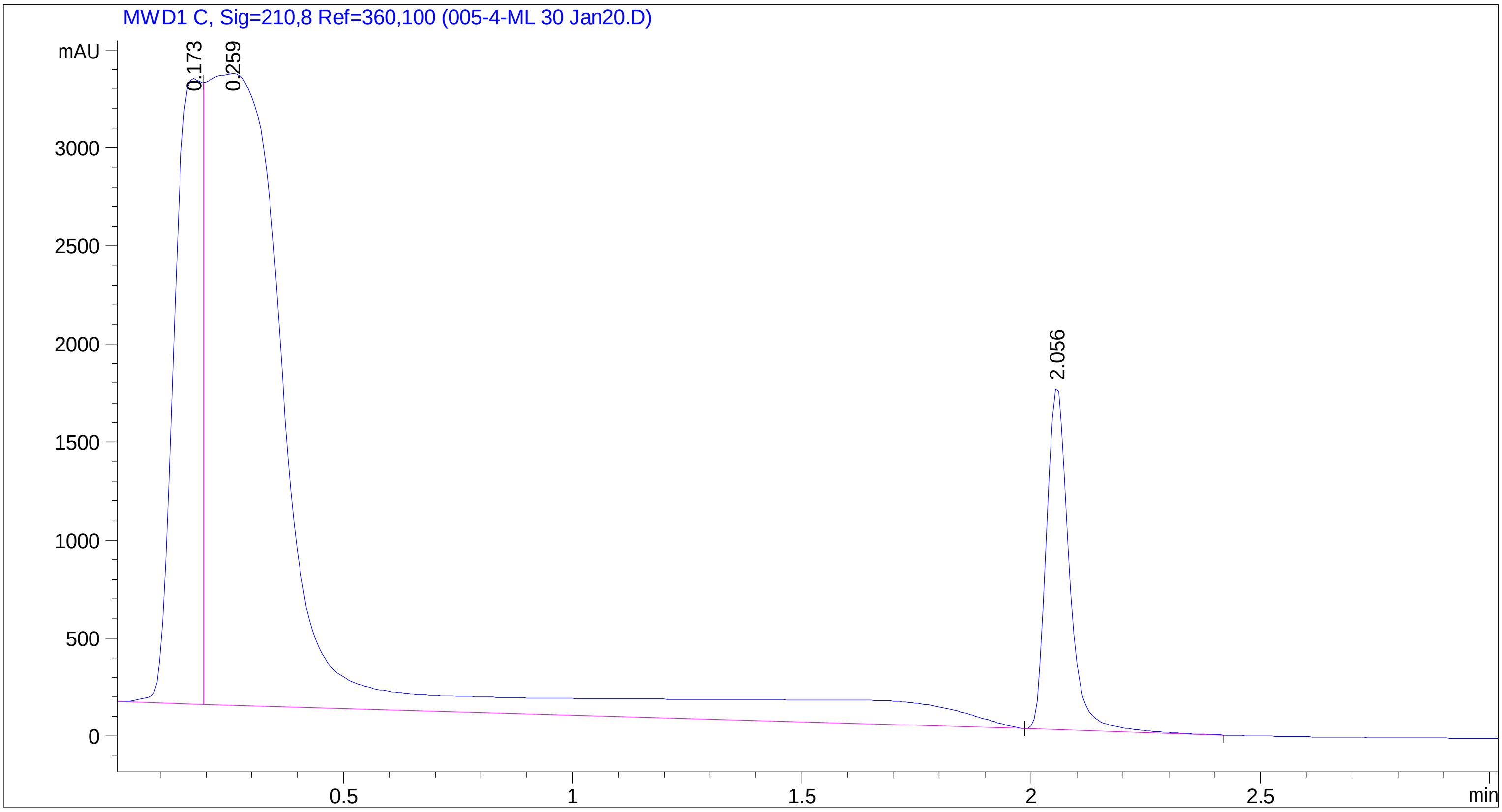

===

Area Percent Report

===

Sorted By : Signal
Multiplier : 1.0000
Dilution : 1.0000
Use Multiplier & Dilution Factor with ISTDs

Signal 1: MWD1 C, Sig=210,8 Ref=360,100

Peak #	RetTime [min]	Type	Width [min]	Area [mAU*s]	Height [mAU]	Area %
1	0.173	BV	0.0643	1.32031e4	3188.51147	21.1229
2	0.259	VB	0.1908	4.32732e4	3219.80103	69.2304
3	2.056	BBA	0.0522	6029.73779	1751.01978	9.6466

Totals : 6.25061e4 8159.33228

===

*** End of Report ***
